# Non-bacterial Thrombotic Endocarditis in a Patient With Squamous Cell Carcinoma of the Lung: Diagnostic Challenges and Management Perspective

**DOI:** 10.7759/cureus.113674

**Published:** 2026-07-30

**Authors:** Narththana Keerthinathan, Kanagasingam Arulmoly, Ramanathan Ramesh

**Affiliations:** 1 General Medicine, Teaching Hospital Batticaloa, Batticaloa, LKA

**Keywords:** cancer associted hypercogulability, marantic endocarditis, non-bacterial thrombotic endocarditis, squamous cell carcinoma of the lung, sterile valvular vegitation, therapeutic anticoagulation

## Abstract

Non-bacterial thrombotic endocarditis (NBTE), also known as marantic endocarditis, is a rare entity characterized by sterile vegetation on cardiac valves. It is typically associated with underlying hypercoagulable conditions, such as a patient with malignancy or autoimmune disease. We report a 70-year-old patient who presented with chronic cough, intermittent fever, progressive dyspnea, and significant weight loss. Imaging revealed left-sided pleural effusion with lung collapse, and further evaluation identified a central bronchial mass consistent with squamous cell carcinoma of the lung with liver metastasis. Transthoracic echocardiography demonstrated a 13-mm vegetation on the anterior mitral valve leaflet, initially raising suspicion for infective endocarditis.

However, multiple blood cultures remained persistently negative, and there was no clinical or radiological evidence of systemic embolization. Despite prolonged empirical antibiotic therapy, vegetation persisted unchanged. In the context of underlying malignancy, a diagnosis of NBTE was established. Anticoagulation was deferred due to recurrent hemoptysis associated with the bronchial tumor. Overall, this case highlights the need for broader diagnostic thinking beyond infective etiologies when evaluating cardiac vegetation, especially in patients with systemic features suggestive of malignancy.

## Introduction

Non-bacterial thrombotic endocarditis (NBTE), also known as marantic endocarditis, is a rare condition characterized by sterile vegetations on the cardiac valves. It is typically associated with underlying hypercoagulable states, particularly in patients with malignancy or autoimmune diseases [1].

Malignancy is a major predisposing factor for NBTE, with the strongest associations observed in mucin-producing adenocarcinomas, including those arising from the lung, ovary, biliary tract, pancreas, breast, and stomach [2]. Malignancy-associated hypercoagulability is not attributable to a single mechanism but rather reflects a complex interplay of multiple pathophysiological processes. These include leukocytosis, thrombocytosis, increased expression of tissue factor and procoagulant phospholipids, and elevated circulating inflammatory markers [3].

Autopsy and echocardiographic studies estimate the incidence of NBTE to range from 0.3% to 9.3% of all endocarditis cases, with a higher prevalence among patients with malignancy, reaching up to 10%-15% in some series [4].

Non-bacterial thrombotic endocarditis is frequently associated with systemic embolic events, including ischemic stroke and renal or splenic infarction. However, cases without embolic manifestations are exceedingly rare. The absence of embolic events poses a significant diagnostic challenge, as the condition may mimic other causes of valvular vegetations, leading to delays in diagnosis and appropriate management [2].

In this case, we describe a 70-year-old man who presented with progressive respiratory distress, intermittent fever, and constitutional symptoms. Diagnostic evaluation confirmed the presence of a primary lung malignancy and probable non-bacterial thrombotic endocarditis associated with the underlying malignancy.

## Case presentation

A 70-year-old man with a known history of atrial fibrillation presented with a 2-month history of cough, intermittent fever, and progressively worsening shortness of breath. He also reported significant unintentional weight loss of approximately 10 kg over the preceding 3 months. His medical history was notable for chronic tobacco smoking and occasional alcohol consumption.

On examination, the patient appeared pale but was not icteric. His vital signs were as follows: blood pressure, 100/60 mmHg; pulse rate, 100 beats per minute with an irregularly irregular rhythm; respiratory rate, 30 breaths per minute; and oxygen saturation, 94% on room air. He was afebrile at presentation. Respiratory examination revealed reduced breath sounds over the left hemithorax with stony dullness to percussion, suggestive of a pleural effusion. Examination of the cardiovascular, abdominal, and neurological systems was otherwise unremarkable.

Laboratory investigations demonstrated mild anemia with normal white blood cell and platelet counts. Inflammatory markers were elevated, with a C-reactive protein (CRP) level of 75 mg/L and an erythrocyte sedimentation rate (ESR) of 55 mm/hour. Apart from mild hyponatremia and mild hypercalcemia, all other laboratory parameters were within normal limits (Table 1).

**Table 1:** On-admission investigations Laboratory investigations reveal moderate anemia with elevated inflammatory markers, indicating an ongoing inflammatory process. There is mild hyponatremia and mild hypercalcemia, while liver function tests, renal function parameters, serum potassium, coagulation profile, procalcitonin, serum LDH, and serum protein are within normal limits. Rheumatoid factor and ANA are negative. Overall, the findings are suggestive of an inflammatory condition associated with anemia. AST: aspartate aminotransferase; ALT: alanine aminotransferase; ANA: antinuclear antibody

INVESTIGATIONS	VALUE	REFERENCE RANGE
WBC	7.72×10^3^/uL	4.00-11.00×10^3^/uL
Hb	9.2×10^3^/uL	12.0-16.0×10^3^/uL
PLT	327×10^3^/uL	150-450×10^3^/uL
Na	133 mmol/l	136-145 mmol/l
Potassium	3.7 mmol/l	3.5-5.1 mmol/l
CRP	75 mg/l	0-5 mg/l
AST	35 U/L	15-37 U/L
ALT	29 U/L	12-78 U/L
Creatinine	66 umol/l	62-115 umol/l
Blood urea	4.0 mmo/l	1.8-6.3 mmol/l
INR	1.06	0.8-1.1
Procalcitonin	0.4 ng/ml	<0.5 ng/ml
ESR	55 mm/1 hr	<20 mm/1hr
Serum LDH	222 U/L	140-280 U/L
Serum protein	71 g/l	64-83 g/l
Serum calcium	2.69 mmol/L	2.1-2.6 mmol/L
Rheumatoid factor	2I U/ml	<8 IU/ml
ANA	6 U/ml	<20 U/ml

**Figure 1:**
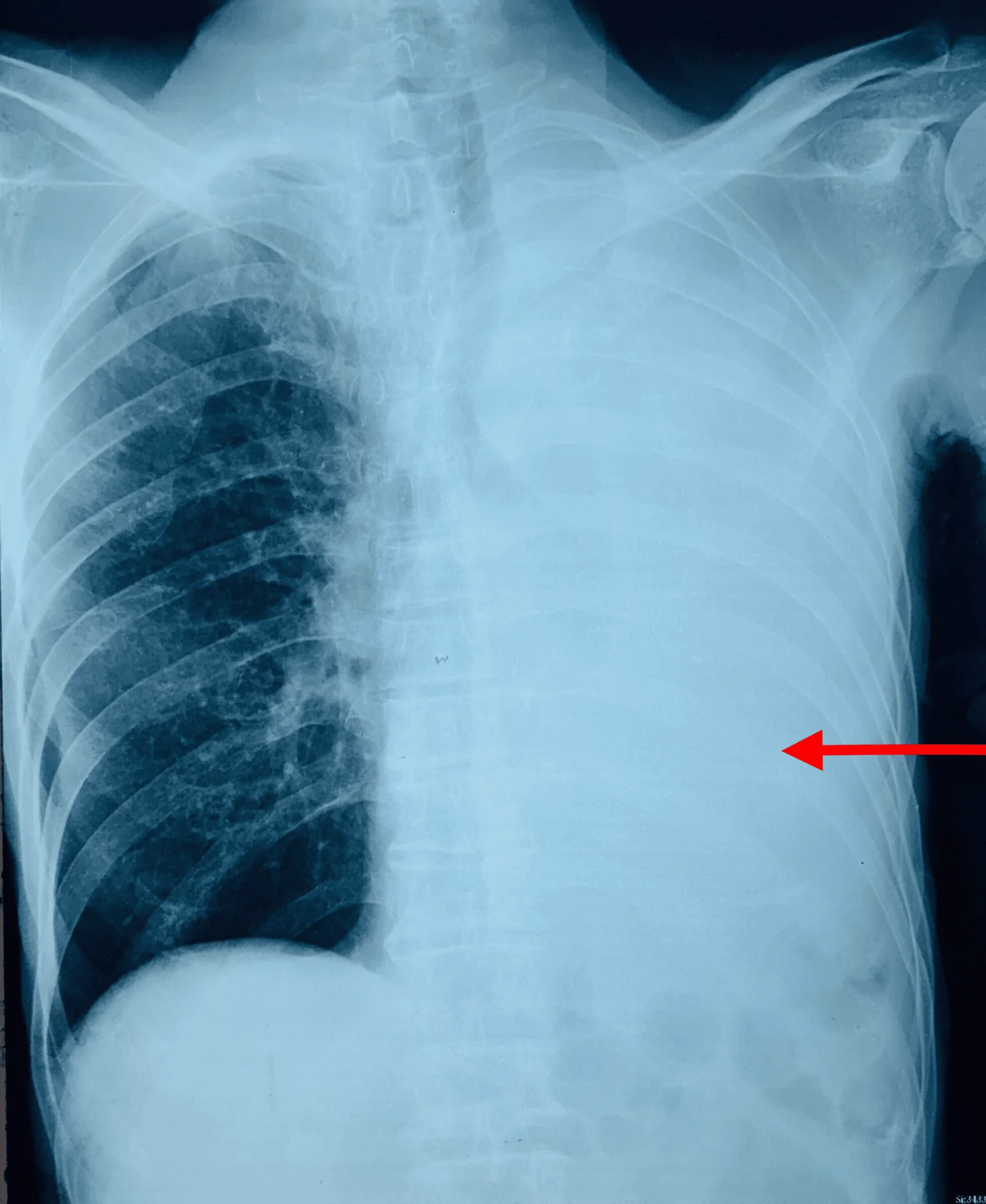
Chest radiography showing massive left-sided lung pleural effusion (red arrow) Posteroanterior chest radiograph demonstrating near-complete opacification of the left hemithorax (red arrow) with contralateral mediastinal shift, consistent with a massive left-sided pleural effusion causing compressive atelectasis of the underlying lung.

Chest X-ray posteroanterior (CXR-PA) view taken on admission demonstrates a massive, left-sided pleural effusion with left lung collapse (Figure 1).

An intercostal chest tube was inserted, and the patient was started on intravenous ceftriaxone for presumed pneumonia with a parapneumonic pleural effusion. Sputum culture, acid-fast bacillus (AFB) smear, and GeneXpert Mycobacterium tuberculosis (MTB)/rifampin (RIF) testing were all negative. Cytological examination of pleural fluid was negative for granulomas and malignant cells. Pleural fluid analysis was consistent with an inflammatory exudative pleural effusion. The pleural fluid glucose level and red blood cell count were within normal limits, with no evidence of significant hemorrhage (Table 2).

**Table 2:** Pleural fluid analysis Pleural fluid analysis revealed elevated protein levels (62 g/L) with normal glucose (5.3 mmol/L) and lactate dehydrogenase (LDH) (173 U/L). In conjunction with a serum protein of 71 g/L and serum LDH of 222 U/L, the fluid satisfies Light's criteria for an exudate [5]. The cellular profile showed 220 lymphocytes/mm³, 440 neutrophils/mm³, and 85 RBCs/mm³, supporting an inflammatory exudative pleural effusion.

Parameters	On-admission value	Reference range
Protein	62g/dl	<25g/l
Glucose	5.3	3.3-5.6mmo/l
Lymphocyte	220/mm^3^	<1000/mm^3^
Neutrophil	440/mm^3^	
RBC	85/mm^3^	<250/mm^3^
LDH	173U/L	88-227U/L

**Figure 2:**
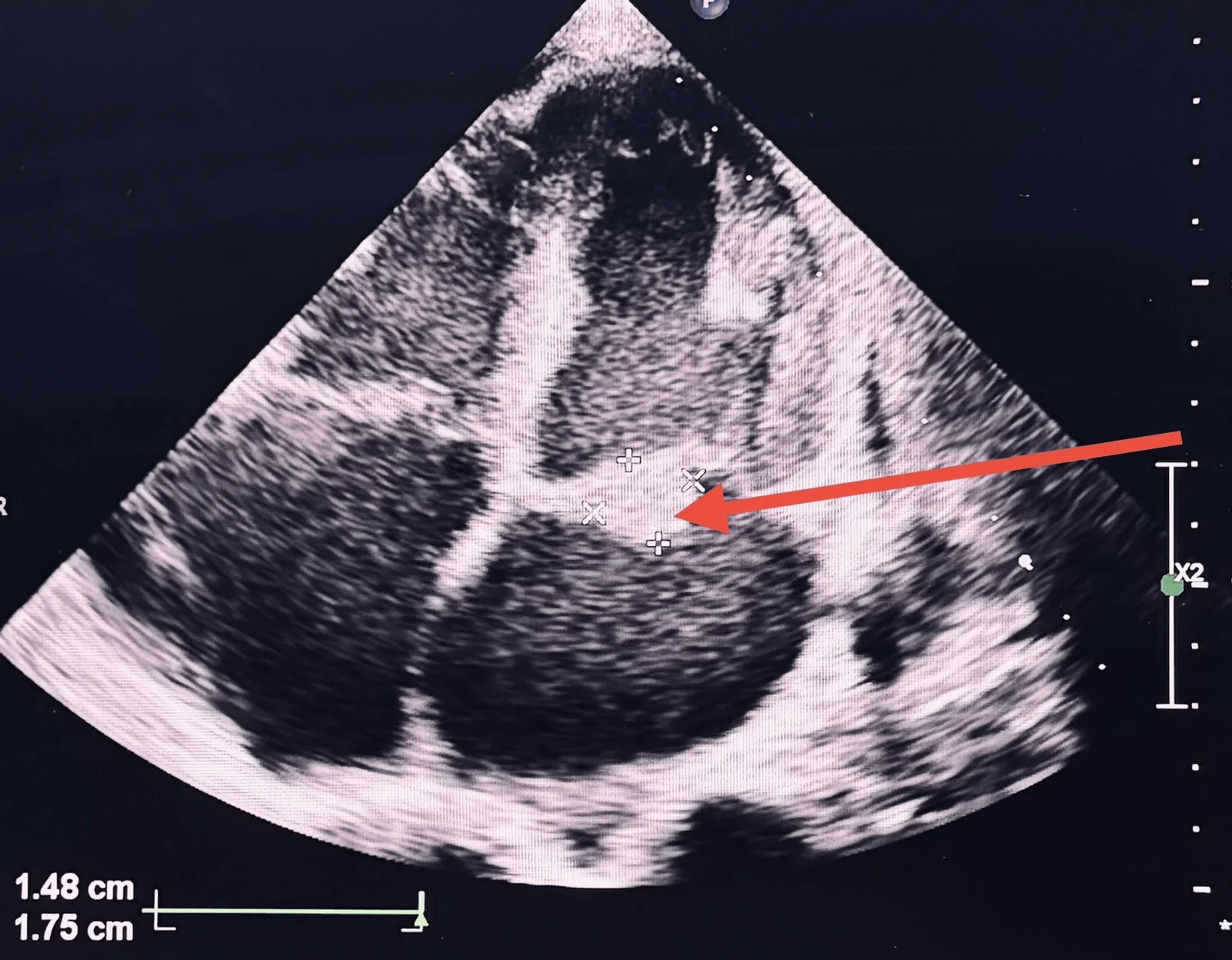
Transthoracic echocardiogram of the anterior mitral valve leaflet vegetation (red arrow) Apical four-chamber transthoracic echocardiographic image showing a pedunculated vegetation arising from the anterior mitral valve leaflet (red arrow), measuring 13 × 14 mm, suggestive of mitral valve endocarditis.

As this patient had a history of prolonged fever with dyspnea, a transthoracic echocardiogram was performed. The echocardiogram demonstrated vegetation on the anterior mitral valve leaflet (Figure 2).

Based on these findings, a diagnosis of infective endocarditis was initially suspected, and the patient was started on intravenous vancomycin after microbiological samples were obtained. An extensive microbiological evaluation, including three sets of blood cultures, urine culture, fungal blood culture, HIV antigen testing, and Venereal Disease Research Laboratory (VDRL) testing, was unremarkable.

Beginning on the second day of admission, the patient developed multiple episodes of hemoptysis, which persisted throughout the hospital stay. Bronchoscopy revealed an actively bleeding mass in the left main bronchus, causing complete obstruction of the left main bronchus. Cytological examination of the bronchial washings did not reveal granulomas or malignant cells.

**Figure 3:**
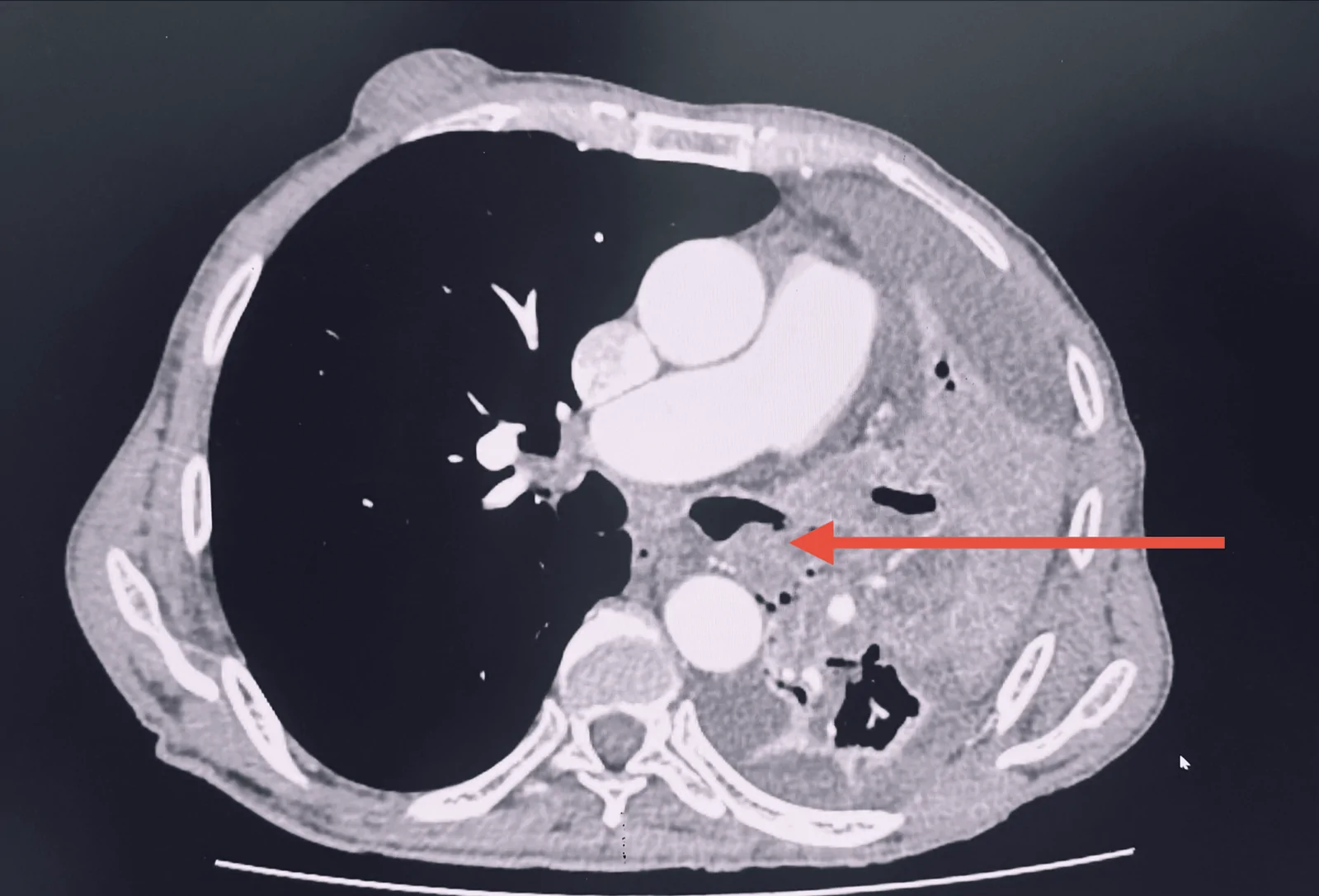
CECT of the chest demonstrating a 5.5*6.5*6.5 cm sized, heterogeneously enhancing, irregular, vague solid and cystic complex mass lesion in the left hilar region (red arrow) Contrast-enhanced computed tomography (CECT) of the chest (axial section) demonstrating a left hilar region mass (red arrow), causing complete obstruction of the left main bronchus and left lung collapse, and compressing the segmental branches of the left pulmonary artery with regional infiltration into mediastinal pleura.

Subsequent contrast-enhanced computed tomography (CECT) of the chest demonstrated a left hilar mass, highly suggestive of central bronchogenic carcinoma, with evidence of mediastinal lymph node involvement (Figure 3). Further evaluation with CECT of the abdomen revealed liver metastases.

Histopathological examination of the biopsy specimen obtained from the left bronchial mass confirmed a moderately differentiated squamous cell carcinoma.

The patient was continued on empirical antibiotic therapy for a total of 39 days because of the initial suspicion of infective endocarditis, particularly in the context of a resource-limited setting where investigations for fastidious organisms, such as *Coxiella burnetii* and *Bartonella* species, were unavailable.

A repeat transthoracic echocardiogram performed at the end of antibiotic therapy demonstrated no significant change in the size of the valvular vegetation. Negative antinuclear antibody (ANA) testing, together with the absence of clinical features suggestive of systemic lupus erythematosus (SLE), made SLE-associated NBTE unlikely. Given the persistently negative blood cultures, the lack of response to prolonged antibiotic therapy, and the presence of advanced malignancy, a diagnosis of probable non-bacterial thrombotic endocarditis (NBTE) was considered.

Following a multidisciplinary discussion involving the cardiology and microbiology teams, antibiotic therapy was discontinued, and the patient was referred for oncological management. Anticoagulation was withheld because of the high risk of bleeding in the setting of ongoing, significant hemoptysis.

The patient required further hospitalization for the management of recurrent hemoptysis. Unfortunately, he declined further oncological treatment and died one month later due to disease progression.

## Discussion

NBTE is a rare condition most commonly observed in patients with advanced malignancy or autoimmune diseases that are associated with a hypercoagulable state.

This patient initially presented with a chronic cough and intermittent fever. Investigations revealed elevated inflammatory markers and a unilateral exudative pleural effusion. Subsequent investigations, including negative pleural fluid TB-PCR, negative sputum AFB smears, negative sputum cultures, negative rheumatoid factor, and negative ANA testing, excluded common infectious and inflammatory causes and increased the suspicion of an underlying malignancy.

Although this patient did not fulfill the modified Duke criteria [6] for a definite diagnosis of infective endocarditis, meeting only one major criterion (valvular vegetation) and one minor criterion (history of fever), the presence of valvular vegetation, elevated inflammatory markers, and the inability to exclude culture-negative infective endocarditis because of limited diagnostic resources justified empirical treatment for infective endocarditis.

This clinical overlap highlights the diagnostic challenge posed by the coexistence of malignancy and raises the possibility of non-bacterial thrombotic endocarditis. It is therefore important to maintain a high index of suspicion for NBTE in patients who are being treated for infective endocarditis [7].

The absence of malignant cells in both the bronchial washings and pleural fluid cytology further complicated the diagnostic workup. This case highlights the importance of maintaining a broad differential diagnosis and pursuing a comprehensive evaluation rather than anchoring to a single initial diagnosis.

The identification of a mitral valve vegetation necessitated consideration of a broad differential diagnosis, including infective vegetation, non-bacterial thrombotic endocarditis, valvular calcification, cardiac myxoma, primary cardiac sarcoma, and metastatic cardiac tumor. Although histopathological confirmation of the valvular lesion was unavailable, the combination of advanced metastatic squamous cell carcinoma, persistently negative blood cultures, failure to fulfill the modified Duke criteria for definite infective endocarditis, lack of regression of the mitral valve vegetation despite prolonged antibiotic therapy, and the absence of an alternative diagnosis strongly supported a diagnosis of probable non-bacterial thrombotic endocarditis.

Because of the highly procoagulant nature of NBTE vegetations and the underlying malignancy, together with the limited therapeutic options once major thromboembolic complications have occurred, the prognosis is poor, with a reported mortality rate of approximately 38%. Most cases reported in the literature are diagnosed postmortem [8].

The management of NBTE is based on two fundamental principles: prompt and effective anticoagulation to reduce the risk of further thromboembolic events, and treatment of the underlying malignancy to eliminate the prothrombotic stimulus [4]. The primary goal of therapy is to prevent systemic embolization, the most common and potentially life-threatening complication of NBTE [9].

In our patient, a multidisciplinary discussion involving cardiology, oncology, and microbiology teams was undertaken to determine the appropriateness of anticoagulation. However, anticoagulation was withheld because of recurrent episodes of significant hemoptysis and bronchoscopic evidence of active bleeding from the bronchial mass. Unfortunately, the patient defaulted from follow-up before anticoagulation could be reconsidered and died from disease progression one month after the diagnosis.

## Conclusions

This case highlights non-bacterial thrombotic endocarditis (NBTE) as an important cancer-associated manifestation of advanced lung carcinoma and a significant diagnostic challenge when valvular vegetations are identified in the absence of evidence of infection. Persistently culture-negative vegetations, lack of response to prolonged antimicrobial therapy, and clinical features suggestive of an underlying malignancy should prompt consideration of NBTE. Early recognition through a multidisciplinary approach facilitates timely evaluation for an occult malignancy, avoids unnecessary prolonged antibiotic exposure, and enables appropriate oncological management. Clinicians should maintain a high index of suspicion for NBTE in patients presenting with unexplained valvular vegetations, particularly in the presence of known or suspected malignancy.
